# Comparative characterization of the gluten and fructan contents of breads from industrial and artisan bakeries: a study of food products in the Spanish market

**DOI:** 10.29219/fnr.v66.8472

**Published:** 2022-06-10

**Authors:** Miriam Marín-Sanz, Susana Sánchez-León, Elena León, Francisco Barro

**Affiliations:** 1 Department of Plant Breeding, Institute of Sustainable Agriculture (IAS), Spanish Council for Scientific Research (CSIC), Córdoba, Spain; 2 IES-Fidiana, Córdoba, Spain

**Keywords:** gluten, fructans, wheat, celiac disease, NCWS

## Abstract

**Background:**

The consumption of wheat/gluten is associated with adverse reactions for human health. Gluten and fructans are identified as the major compounds triggering and worsening adverse reactions to wheat, which are increasing, and as a consequence, avoidance of gluten/wheat is the common strategy of many individuals of the western population. Although bread is a product of daily consumption, there is a lack of information on the gluten and fructan contents and the influence of artisanal or industrial processes.

**Objective:**

The aim of this study is to carry out a comparative characterization between artisan bakeries and hypermarkets in Spain for gluten and fructan contents in daily sold breads.

**Design:**

A total of 48 types of bread highly consumed in Spain sold in artisan bakeries (long fermentation) and hypermarkets (short fermentations) were selected for comparing the gluten and fructan contents. Methods such as reverse phase-high performance liquid chromatography (RP-HPLC), R5 monoclonal antibody (moAb), and fructans protocols were used for the quantification of these compounds.

**Results:**

Great variation for the content of gluten and fructans has been found between all bread categories. Although breads produced using long fermentation (artisan bakeries) contain significantly lower gluten, they have higher fructans than those using short fermentations (hypermarkets). Durum wheat breads had the lowest content of gluten. Moreover, spelt breads from artisan bakeries had the lowest content of fructans but not those from hypermarkets.

**Discussion:**

In this study, we report the comparative characterizarion of the breads of the Spanish market. These food products presented variation in the amount of gluten and fructans, ligated in most of the cases to the nature of the providers: artisan bakeries against hypermarkets. Depending on the type of bread, the differences for the daily consumption of gluten and fructan can be 4.5 and 20 times, respectively.

**Conclusions:**

We found strong differences for gluten and fructan contents among breads. These information may contribute to designing strategies to improve the management of gluten and fructans in bread.

## Popular scientific summary

- This study reports that bread products sold in the Spanish market present strong differences for the content of gluten and fructan – the main compounds related to celiac disease and non-celiac wheat sensitivity. Industrial and artisan bakeries were compared.
- This information is important for daily consumed food in the context of concern about wheat-related disorders and, therefore, for designing new strategies for improving the management of these compounds in breads.

Bread is the main staple food in western countries, providing carbohydrates, energy, and proteins for the human diet (1). Although wheat is the basis of bread due to the unique viscoelastic properties of the gluten proteins, other cereals such as rye, barley, or spelt or a mix of them can be used to produce many popular bread types. The consumption of wheat-derived products is also increasing in countries, where it is not climatically adapted, particularly in countries adopting a ‘western lifestyle’.

The consumption of wheat is also associated with three adverse reactions for human health: celiac disease (CD), wheat allergy (WA), and non-celiac wheat sensitivity (NCWS). CD and WA are immunological diseases with known underlying mechanisms. In CD, the response is triggered by human leukocyte antigen (HLA) HLA-DQ2 and HLA-DQ8-presented peptides that are recognized by CD4 T cells (2). WA is an IgE-mediated disease, which includes wheat-dependent exercise-induced anaphylaxis (WDEIA) and Baker’s asthma. For CD, gluten proteins play a major role, and the gliadin fraction of gluten contains the most important epitopes triggering the immunogenic response (3). In contrast, the cause and mechanisms of NCWS are still unclear. Although gluten was initially thought to be the causative agent of the disease, subsequent studies have shown a predominant role of FODMAPs (fermentable oligosaccharides, disaccharides, monosaccharides, and polyols) (4, 5), particularly fructans, which are hydrolyzed partially in the intestine causing symptoms as bloating and abdominal pain (5), and α-amylase/trypsin inhibitors (ATIs) (6).

In western countries, the mean frequency of CD is about 1% with a range of variation depending on the region. In contrast, the prevalence of NCWS is still unknown due to cases of self-diagnoses and the lack of standardized diagnostic criteria or biomarkers, but it is estimated to be 5–10 times higher than that of CD and WA (7). Evidences support that CD has increased in recent years, both in Europe and in the United States (8), and in addition to wider use of the serological test for screening CD (8), other contributing factors have been proposed: the increasing of nitrogen (N) fertilization in the last five decades for cereal crops that have been correlated with the increment of immunogenic gliadins of wheat (9); the breeding and expansion of wheat varieties, with enhanced breadmaking quality but may harbor highly immunogenic peptides (10); or the expansion of industrial baking techniques with short fermentation times that minimize proteolytic process, leading to highly immunogenic-undigested peptides (11).

As a consequence of the increase in wheat-related pathologies, avoidance of gluten/wheat is the common strategy of many individuals of the western population. This leads to a loss of confidence in one of our most important staple foods, bread, which might jeopardize the agriculture and bakery industries and further be detrimental toward a high-fiber diet. In addition, following a gluten-free diet (GFD) can suppose a nutritional imbalance (12).

For artisan bakeries, to produce traditional bread, fermenting the sourdough at a very low temperature for a long time is essential (13), while the industrial process uses dried sourdoughs as a convenient ingredient in bakery premixes (14) and short fermentation time. In addition, the consumption of industrial and artisan bread varies greatly depending on the region and country. In Spain, 19% of the bread production is represented by the industrial sector, while in the United Kingdom, it is 80% (14). Sourdough fermentation has been suggested to provide low gluten wheat-based products through proteolytic degradation of wheat grain proteins (15), and also to reduce the fructan content of bread (16). Although the consumption of bread is inevitably associated with the intake of compounds related to these pathologies, there is a concern among consumers that they are consuming excessive amounts of immunogenic compounds. In this work, we have compared the immunogenic potential of different types of bread, daily consumed in Spain, provided by artisan bakeries and hypermarkets. This knowledge will contribute to designing strategies to better manage the content of gluten and fructans in bread, either using plant breeding, developing hypoallergenic varieties, or from the baking processes, gaining insight into which processes favor the degradation of these compounds without penalising quality.

## Materials and methods

Breads were obtained from hypermarkets and artisan bakeries located in Córdoba, Sevilla, Madrid, Barcelona, and Zaragoza. All breads from artisan bakeries were made using sourdough and long fermentation process (minimum of 12 h fermentation), while breads from hypermarkets do not use of sourdough and long fermentation process.

For each piece of bread, four slices were cut, weighted, and lyophilized for calculating the relative moisture of their respective breads. The slices of every bread were grinded in a cyclone mill together.

### Total protein and starch content of breads

The protein content of the bread was determined using the Dumas (%*N* × 6.25) method, while the starch content was determined according to the polarimetric method by Eurofins (Eurofins Análisis Alimentario SL, Madrid, Spain, www.eurofins.es). The total content of protein and starch per bread sample was expressed on dry weight (DW).

### Total prolamin extraction and quantification by reverse phase-high performance liquid chromatography

#### Extraction of prolamins

Total prolamin was extracted in quintuplicate from each bread using the glutenin extraction protocol as described in Pistón et al. (17). Briefly, total prolamin was extracted from the insoluble pellet stepwise three times with 670 μL of glutenin extraction buffer C1 (50% [v/v] 1-propanol, 2 M urea, 0.05 M Tris–HCl [pH 7.5], and 2% [w/v] dithiothreitol (DTT)), vortexing for 1 min at room temperature (RT), and incubation for 30 min at 60°C in an orbital shaker. Then, samples were centrifuged for 15 min at 6,000 × g at 24°C, and supernatants collected and mixed in a single tube.

For gliadin and glutenin extraction of T80 spelt flour, we used buffer B1 and C1, respectively. Gliadins were extracted stepwise three times in buffer B1, 670 μL of 60% (v/v) ethanol. After centrifugation, the supernatants were collected and mixed together. This fraction was resuspended in C1 buffer for reverse phase-high performance liquid chromatography (RP-HPLC) quantification. The insoluble pellet was used for glutenin extraction as described previously for total prolamins.

#### Quantification of prolamins by RP-HPLC

Prior to RP-HPLC, a 700 μL of protein extracts was centrifuged through a 0.45 µm pore Corning^®^ Costar^®^ Spin-X nylon filter. Volumes of prolamin extracts (60 μL) were applied to a 300SB-C8 reverse phase analytical column (4.6 × 250 mm, 5 µm particle size, and 300 Å pore size; Agilent Technologies) using a 1,200 series high-resolution liquid chromatography quaternary system (Agilent Technologies) with a DAD UV-V detector, as described in Refs. (18) and (17). The elution system consisted of deionized water (A) and acetonitrile (B), both containing 0.1% (v/v) trichloroacetic acid. The proteins were eluted with a linear gradient from 24% B to 56% B in 60 min, at a flow rate of 0.5 mL/min at 50°C. Absorbance was monitored using the DAD UV-V module at 210 nm, and integration was automatically handled by the RP-HPLC system software with some manual adjustments. Absolute amounts of prolamins were determined using bovine serum albumin (BSA, 98% BSA, fraction V, Sigma-Aldrich, St Louis, MO, USA, cat No. A3294) as a protein standard.

### Determination of gluten by R5 monoclonal antibody

Gluten content in parts per million (ppm) was determined using the RIDASCREEN^®^ Gliadin competitive (R-Biopharm AG, Darmstadt, Germany) enzyme-linked immunosorbent assays (ELISA) kit, using the monoclonal antibody R5 (19) at Centro Nacional de Biotecnología (CNB, www.cnb.csic.es). Three independent replicates were carried out for each sample.

### Fructan content

The fructan content per DW was determined in duplicate using 150 mg of sample by the Megazyme commercial kit K-FRUC (www.megazyme.com) following the manufacturer assay procedure. To express the fructan content per DW, the moisture of each sample was determined.

### Statistical analysis

For statistical analysis, R v. 3.6.1 (20) was used. Statistical differences of bread components between samples were determined by the ANOVA, *t*-test, Mann–Whitney test, or Kruskal–Wallis test. We performed a principal component analysis (PCA) including all the variables measured in the present work to evaluate their contribution to the model variance. The libraries FactoMineR (21) and Factoextra (22) were used for PCA analysis and graphical output, respectively.

## Results

In this study, the immunogenic potential of 48 different types of bread commercialized in Spain was evaluated (Supplementary Table 1). Four categories of breads have been established: rye breads, spelt breads, whole grain (or whole meal) breads, and breads mostly made using bread wheat flour, but which may contain other cereals. Finally, a projection of the daily intake of immunogenic compounds (gluten and fructans) has been made based on the type of bread.

### Prolamin quantification

Gluten proteins (also called prolamins) are divided into two different fractions, the gliadins and the glutenins (23), which can be extracted from the flour and quantified by RP-HPLC. However, the extraction and quantification of the prolamin fractions in processed foods, such as bread, is more complex than that performed on flour because of monomeric fraction comprising the gliadins becomes part of the polymeric fraction together with the glutenins as a consequence of the heat-induced formation of interchain disulphide bonds (18). To differentiate between the different prolamin fractions in bread, gliadins and glutenins were extracted from the flour of T80 spelt, subject to RP-HPLC separation, and the distribution of gliadins and glutenins throughout the retention time compared to that of the total prolamin extracts from bread chromatograms obtained in previous works (24). The limits between two clear protein regions, named in this work as Protein 1 and Protein 2, and encompassing the ω-gliadins and High Molecular Weight (HMW)-glutenins, and α-gliadins, γ-gliadins, and Low Molecular Weight (LMW)-glutenins, respectively (Supplementary Fig. 1), were established in bread (24). The Protein 2 fraction corresponds to S-rich prolamin proteins, while Protein 1 fraction corresponds to S-poor gliadins and HMW (25).

### Gluten and fructans contents

Nine different breads containing rye were compared (Table 1). The content of rye flour varies between 32 and 64% for hypermarket breads, while it was 100% in breads from artisan bakeries, except for one which contains 25% of rye flour (Table 1). The RP-HPLC profiles made it possible to differentiate the variable amount of rye in this type of bread: most of the hypermarket rye breads showed a profile similar to that of only 25% rye in its composition (Supplementary Fig. 2). The maximum values for gluten and fructan contents were, respectively, 155,604 ppm and 2.22 g/100 g DW, which correspond, respectively, to hypermarkets and artisan bakeries. In fact, the fructan content was higher in all breads from artisan bakeries, except for the bread with 25% of rye flour (Table 1).

**Table 1:** Mean of gluten and fructan contents for hypermarkets and local bakery rye breads

Bread name	Source	Rye (%)	Water (%)	Protein 1 (µg/mg flour)		Protein 2 (µg/mg flour)		Gluten (ppm)		Fructans (g/100 g DW)	
ryeH01	Hypermarkets	64	36.6	5.7	e	27.8	e	131,286	b	0.39	c
ryeH02	Hypermarkets	37	35.7	10.5	ab	**72.2**	**a**	108,222	d	0.48	c
ryeH03	Hypermarkets	32	41.0	7.6	cde	49.8	c	123,211	bcd	0.93	abc
ryeH04	Hypermarkets	53	29.1	**11.2**	**a**	59.3	b	**155,604**	**a**	0.69	bc
ryeH05	Hypermarkets	37	33.6	6.9	de	49.4	c	121,351	bcd	0.85	abc
ryeLB06	Local bakery	100	38.3	7.0	de	25.0	e	113,960	cd	1.59	ab
ryeLB07	Local bakery	100	41.7	8.8	bcd	45.5	cd	154,217	a	**2.22**	**a**
ryeLB08	Local bakery	100	45.8	9.6	abc	42.8	d	130,518	bc	1.75	ab
ryeLB09	Local bakery	25	40.3	7.9	cd	65.9	a	85,890	e	0.99	bc

Statistical differences were established by ANOVA and Kruskal–Wallis for parametric and non-parametric data, respectively. Bread with the highest component content is indicated in bold. For multiple comparisons, the Tukey test and Dunn test were performed for parametric and non-parametric data, respectively. According to the list of ingredients, common bread wheat flour is added to complete 100% flour composition. Gluten (ppm) was determined by R5 monoclonal antibody.

The percentage of spelt flour was 100 in all artisan bakery breads, but it varied greatly among the hypermarket breads, from as low as 11% to a 60% for the highest one (Table 2). One hypermarket spelt bread had the highest values for Protein 1 and Protein 2, and for the gluten (151,967 ppm) content (Table 2). The highest fructan content corresponded to a hypermarket bread with 0.43 g/100 g DW, the one containing the lowest percentage of spelt flour (Table 2).

**Table 2:** Mean of total gluten and fructan contents for hypermarkets and local bakery spelt breads

Bread name	Source	Spelt (%)	Water (%)	Protein 1 (µg/mg flour)		Protein 2 (µg/mg flour)		Gluten (ppm)		Fructans (g/100 g DW)	
speltH01	Hypermarkets	25	29.9	11.7	ab	82.0	c	139,988	ab	0.33	a
speltH02	Hypermarkets	24	34.2	9.7	bcd	81.6	c	112,460	abc	0.35	a
speltH03	Hypermarkets	11	32.9	10.9	abc	85.1	bc	105,577	bc	**0.43**	**a**
speltH04	Hypermarkets	60	33.3	**12.9**	**a**	**107.7**	**a**	**151,967**	**a**	0.15	a
speltLB05	Local bakery	100	37.2	8.4	d	80.0	c	83,563	d	0.18	a
speltLB06	Local bakery	100	38.0	12.4	a	96.6	ab	100,886	c	0.23	a
speltLB07	Local bakery	100	34.1	7.7	d	82.9	bc	94,776	cd	0.19	a
speltLB08	Local bakery	100	40.1	8.6	cd	82.3	c	81,602	d	0.10	a

Statistical differences were established by ANOVA and Kruskal–Wallis for parametric and non-parametric data, respectively. Bread with the highest component content is indicated in bold. For multiple comparisons, the Tukey test and Dunn test were performed for parametric and non-parametric data, respectively. According to the list of ingredients, common bread wheat flour is added to complete 100% flour composition. Gluten (ppm) was determined by R5 monoclonal antibody.

Only three breads were made using 100% whole grain flour, whereas the others were reconstituted by adding wheat bran (Table 3). For Protein 1 and Protein 2 fractions, one local bakery had the highest value, more similar to those of the whole grain breads from hypermarkets (Table 3). For gluten and fructan contents, the highest values were 109,707 ppm and 1.12 g/100 g DW, respectively, which correspond to a hypermarket and artisan bakery, respectively (Table 3). As shown, for both gluten and fructan contents, there were significant differences between the different types of breads.

**Table 3:** Mean of gluten and fructan contents for hypermarkets and local bakery whole grain breads

Bread name	Source	Whole grain (%)	Water (%)	Protein 1 (µg/mg flour)		Protein 2 (µg/mg flour)		Gluten (ppm)		Fructan (g/100 g DW)	
wholegrainH01	Hypermarkets	7	28.2	10.5	bc	83.2	ab	87,395	cd	0.15	c
wholegrainH02	Hypermarkets	100	33.7	9.6	c	82.8	ab	99,788	ab	0.28	bc
wholegrainH03	Hypermarkets	NL	32.3	10.4	bc	81.0	bc	106,879	ab	0.33	bc
wholegrainH04	Hypermarkets	6	28.7	11.9	ab	82.0	ab	**109,707**	**a**	0.32	bc
wholegrainH05	Hypermarkets	NL	30.6	8.3	cd	76.3	cd	89,533	c	0.35	bc
wholegrainLB06	Local bakery	100	41.6	9.7	bc	59.7	d	84,795	cd	0.68	abc
wholegrainLB07	Local bakery	100	41.3	7.0	d	72.4	d	59,431	d	**1.12**	**a**
wholegrainLB08	Local bakery	75	41.2	**12.9**	**a**	**87.7**	**a**	93,923	bc	0.74	ab

Statistical differences were established by ANOVA and Kruskal–Wallis for parametric and non-parametric data, respectively. Bread with the highest component content is indicated in bold. For multiple comparisons, the Tukey test and Dunn test were performed for parametric and non-parametric data, respectively. According to the list of ingredients, common bread wheat flour is added to complete 100% composition. Gluten (ppm) was determined by R5 monoclonal antibody.

Finally, the wheat-based category included breads from bread wheat flour, but there are also breads made using other cereal flour. The most common are those containing durum wheat, particularly in artisan bakeries, the percentage of which varies from 5 to 100%. In this category, the highest values for Protein 1, Protein 2, gluten, and fructans correspond to breads produced in artisan bakeries. Interestingly, one artisan bread had the highest content of gluten (ppm) and Protein 2 (Table 4). However, two breads from artisan bakeries, one made using 100% durum wheat flour and the other using 40% tritordeum flour, showed the lowest values for gluten content.

**Table 4:** Mean of gluten and fructan contents for hypermarkets and local bakery wheat-based breads

Bread name	Source	Other cereals (%)	Water (%)	Protein 1 (µg/mg flour)		Protein 2 (µg/mg flour)		Gluten (ppm)		Fructan (g/100 g DW)	
wheatH01	Hypermarkets	Rye (NL)	25.8	9.1	abcdefg	73.4	efgh	89,335	cdef	0.27	de
wheatH02	Hypermarkets	Rye (NL)	31.2	11.3	ab	88.8	ab	104,696	abc	0.45	abcde
wheatH03	Hypermarkets	NA	27.6	10.4	abc	83.3	abc	111,581	ab	0.41	abcde
wheatH04	Hypermarkets	NA	30.4	9.7	abcde	80.0	bcde	98,261	abc	0.32	cde
wheatH05	Hypermarkets	NA	31.9	8.7	bcdefgh	80.9	abcd	99,738	abc	0.38	bcde
wheatH06	Hypermarkets	Barley (NL)	34.5	8.4	cdefghi	72.0	gh	78,216	defg	0.59	abcd
wheatH07	Hypermarkets	Durum wheat (NL)	23.7	9.8	abcde	80.4	bcd	100,114	abc	0.28	cde
wheatH08	Hypermarkets	NA	29.3	9.5	abcdef	80.3	bcd	89,080	cdef	0.35	bcde
wheatH09	Hypermarkets	NA	32.0	8.0	defghi	71.8	fgh	101,582	abc	0.51	abcde
wheatH10	Hypermarkets	NA	28.8	10.1	abcd	78.6	cdef	92,468	bcde	0.19	e
wheatLB11	Local bakery	Rye (9%)	41.5	11.8	ab	**91.8**	**a**	**113,275**	**a**	0.46	abcde
wheatLB12	Local bakery	NA	38.8	7.0	ghi	71.2	gh	92,752	bcde	0.69	ab
wheatLB13	Local bakery	NA	38.3	**15.1**	**a**	84.0	abc	97,623	abcd	0.29	cde
wheatLB14	Local bakery	Durum wheat (100%)	36.0	5.3	i	88.8	ab	33,626	j	0.39	bcde
wheatLB15	Local bakery	Durum wheat (5%)	33.8	6.5	hi	73.4	fgh	69,792	fgh	0.46	abcde
wheatLB16	Local bakery	Durum wheat (5%)	39.4	7.7	efghi	77.1	cdefg	73,317	efgh	0.39	bcde
wheatLB17	Local bakery	Durum wheat (20%)	35.4	9.7	abcdef	81.5	abcd	72,485	fgh	0.38	bcde
wheatLB18	Local bakery	Tritordeum (40%)	31.1	5.9	i	78.8	cdef	44,349	ij	**0.77**	**a**
wheatLB19	Local bakery	Durum wheat (50%)	38.4	7.9	defghi	77.2	cdefg	53,820	hi	0.51	abcde
wheatLB20	Local bakery	NA	36.2	7.4	fghi	73.5	efgh	62,387	ghi	0.62	abcd
wheatLB21	Local bakery	NA	31.3	7.6	efghi	75.4	defgh	77,459	efg	0.65	abc
wheatLB22	Local bakery	NA	33.9	8.8	bcdefg	78.6	cdef	71,344	fgh	0.45	abcde
wheatLB23	Local bakery	NA	30.8	7.8	defghi	61.9	h	69,102	gh	0.49	abcde

Statistical differences were established by ANOVA and Kruskal–Wallis for parametric and non-parametric data, respectively. Bread with the highest component content is indicated in bold. For multiple comparisons, the Tukey test and Dunn test were performed for parametric and non-parametric data, respectively. According to the list of ingredients, common bread wheat flour is added to complete 100% composition. Gluten (ppm) was determined by R5 monoclonal antibody.

**Fig. 1:**
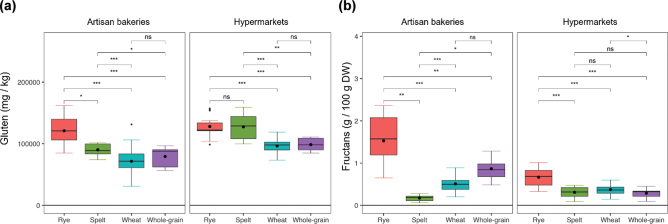
(a) Gluten (mg/kg) and (b) fructan (g/100 g DW) contents in rye, spelt, whole grain, and wheat-based breads from artisan bakeries and hypermarkets. *, *P* < 0.05; **, *P* < 0.01; ***, *P* < 0.001; ns, no significant; DW, dry weight. The black dot indicates the mean. Statistical significances were calculated by *t*-test and Mann–Whitney test for parametric and non-parametric data, respectively.

**Fig. 2:**
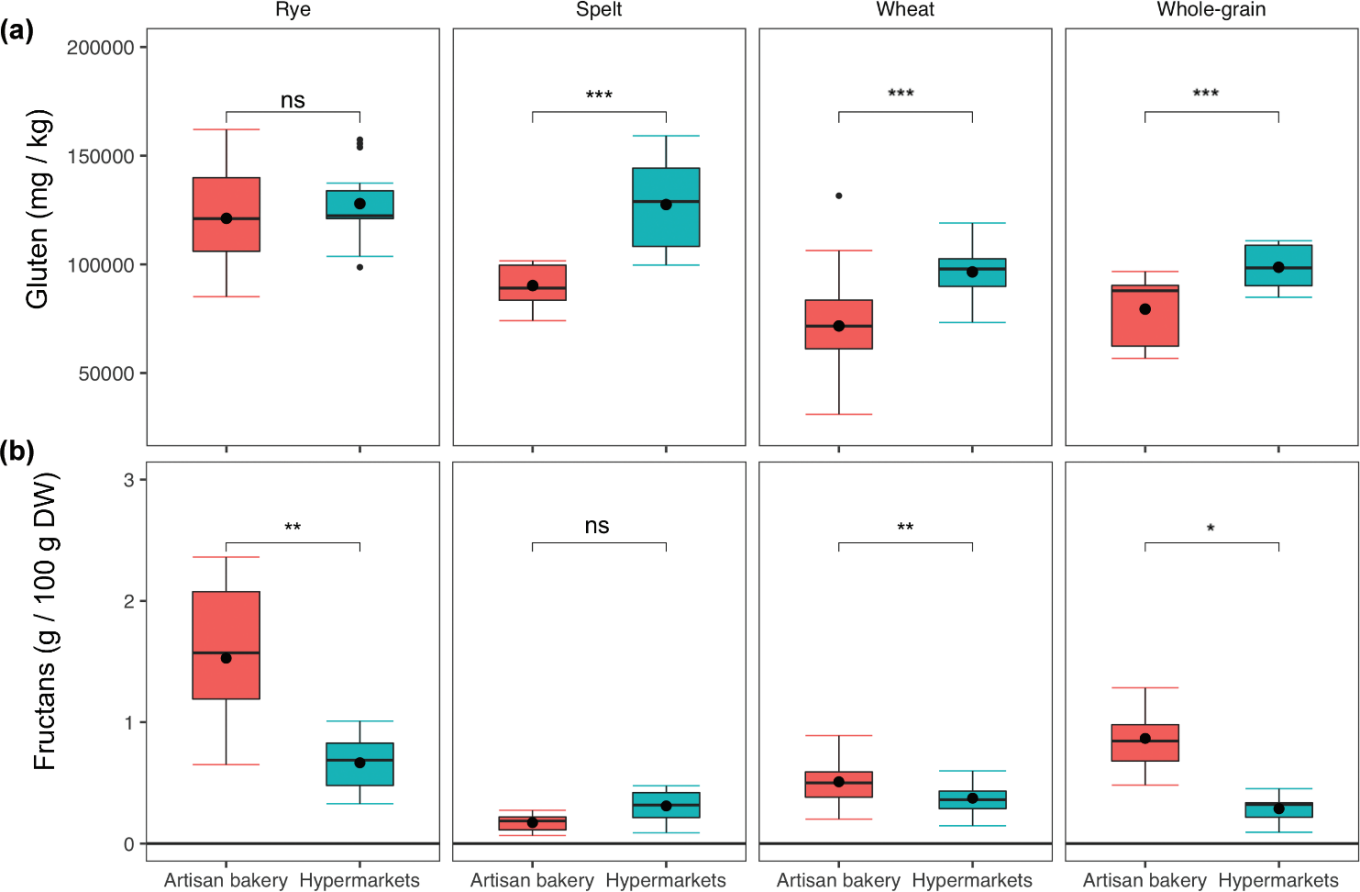
Comparison of breads from artisan bakeries and hypermarkets in terms of gluten (a) and fructan contents (b). Statistically significant differences: ‘***’, ‘**’, and ‘*’, at 0.001, 0.01, and 0.05 levels, respectively; ns, no significant; DW, dry weight. The black dot indicates the mean. Statistical significances were calculated by *t*-test and Mann–Whitney test for parametric and non-parametric data, respectively.

The comparison of gluten (ppm) and fructan contents within each group of bakeries and the comparison between artisan bakeries and hypermarkets are shown in Figs. 1 and 2, respectively. For artisan bakeries, rye breads showed the highest gluten content by the R5 moAb analysis method, with significant differences to all other bread types, which is discussed below. Interestingly, there were non-significant differences between wheat and whole grain breads (Fig. 1). In contrast, for hypermarkets, no differences were found between rye and spelt breads, and there were significant differences between spelt and whole grain breads, with a higher gluten content in spelt breads. As for the artisan bakeries, there were no significant differences between whole grain and wheat breads. Overall, except for rye breads, the gluten content of spelt, whole grain, and wheat breads was significantly higher in breads sold in hypermarkets (Fig. 2).

With regard to fructan content, all types of bread from the artisan bakeries were significantly different from each other, except between wheat and whole-grain breads. Rye breads contained the most fructans, whereas spelt breads contained the least (Fig. 1). In contrast, breads from hypermarkets showed less variability, and only breads containing rye were significantly different from the rest, in addition to the significant differences shown between wheat and whole-grain breads. Overall, except for spelt breads, the fructan content was significantly higher in breads from artisan bakeries than that from hypermarkets (Fig. 2).

### Total protein and starch of breads

The content of total protein was not statistically different between hypermarkets’ and artisan bakeries’ breads (Supplementary Fig. 3). In general, the total protein content was lower for rye breads and reaches higher values for spelt and whole grain breads (Supplementary Fig. 3). Wheat-based breads have lower total protein content than the rest, except for one that contains about 15% of protein (Supplementary Fig. 3).

As for total protein content, total starch was not statistically different between hypermarkets’ and artisan bakeries’ breads (Supplementary Fig. 3). One local bakery whole grain bread stands out with the highest starch content between breads (Supplementary Fig. 3). Interestingly, all wheat-based breads of hypermarkets beat the mean value, whereas this did not occur in wheat breads of artisan bakeries (Supplementary Fig. 3).

### Correlation between prolamin content and gluten content

**Fig. 3:**
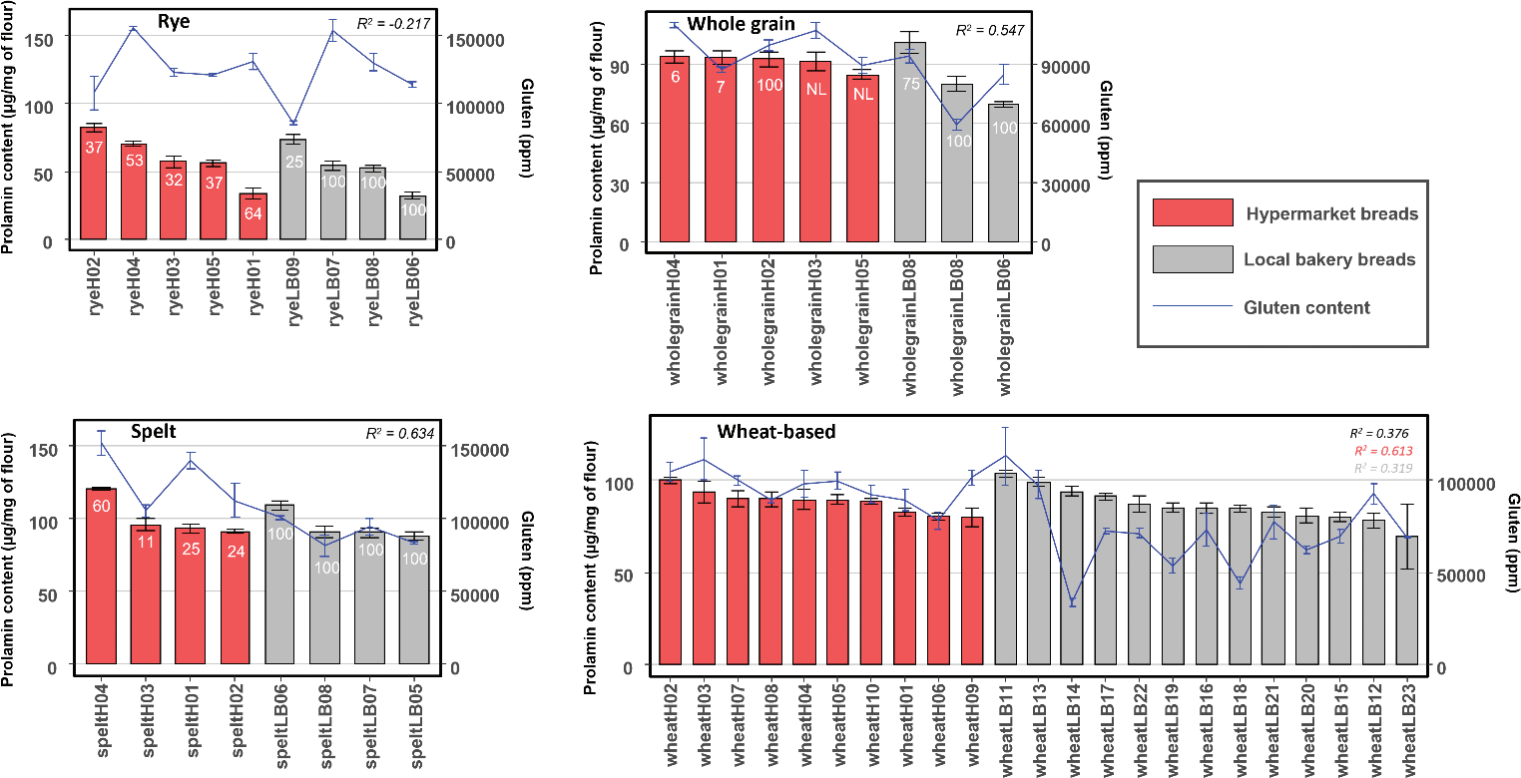
Correlation between the prolamin and gluten by the R5 moAb content of rye, spelt, whole grain, and wheat-based breads from artisan bakeries and hypermarkets. Bars indicate the standard deviation for replicates. *R*^2^ for each type of bread was calculated by the Pearson correlation test. *R*^2^ was also calculated for wheat-based bread from artisan bakeries (grey) and hypermarkets (red) separately.

Correlations for prolamin content determined by RP-HPLC and gluten content determined by monoclonal antibody were studied (Fig. 3). The prolamin content was reassembled considering protein fractions 1 and 2. The prolamin and gluten content (ppm) scales have been adjusted to be comparable in Fig. 3 (100 µg of prolamin/mg of flour = 100,000 ppm of gluten). As shown, for rye-containing breads, there is a poor fit between the prolamin content and the gluten content in ppm (*R*^2^ = −0.217 by Pearson correlation analysis). In the case of spelt breads, the adjustment is much better (*R*^2^ = 0.634), particularly for artisan breads (Fig. 3). For whole grain breads, the fitting (*R*^2^ = 0.547) is also better than for rye breads but slightly lower than that for spelt breads. Finally, for wheat-based breads, the prolamin content and the gluten content showed lower fitting (*R*^2^ = 0.376) than for spelt and whole grain breads. Interestingly, for wheat-based breads, the fitting was better for hypermarket (*R*^2^ = 0.613) than for artisan bakeries (*R*^2^ = 0.319), being lower those made with 100% and 50% of durum wheat, and 40% of tritordeum, respectively (Fig. 3 and Table 4).

### Principal components analysis (PCA)

**Fig. 4:**
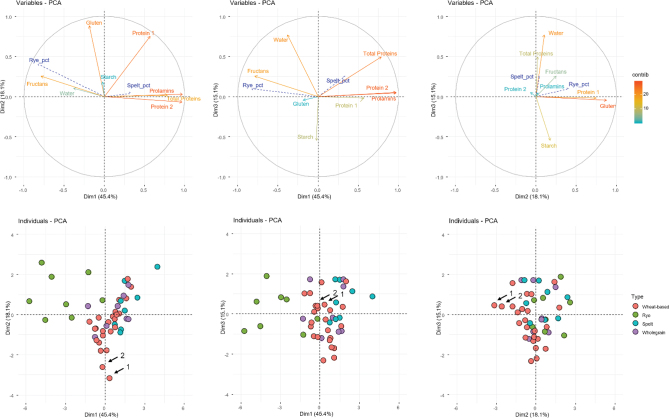
Principal components analysis (PCA). 1) First and second dimensions, 2) first and third dimensions, and 3) second and third dimensions plots of the (up) variables in a color gradient according to the contribution to the variability and supplementary variables in blue and (down) individual breads colored according the generic type they are sold. Variables Protein 1 and ppm contribute almost only to second dimension. Breads pointed with arrows, the lowest immunotoxic ones, are made of (1) 100% durum wheat and of (2) 40% tritordeum. Pct, percentage; Dim, dimension; contrib, contribution.

A PCA was carried out to understand the factors contributing to the variability of gluten and fructan contents in the breads. In Fig. 4, variables in blue indicate supplementary variables that were not taken into account in the analysis (% of rye and spelt of breads). One of the variables that contributes the most to the variance of the model was the prolamin content, mainly Protein 2 fraction that comprises α-, γ-gliadins, and LMW glutenins, followed by the gluten content by R5 moAb (ppm), fructan, and Protein 1 fraction, which comprises the ω-gliadins and HMW glutenins (Fig. 4). As mentioned above, the PCA analysis confirmed the low correlation between the gluten content by R5 moAb and the prolamin content determined by RP-HPLC for the complete set of breads (Fig. 4).

In general, wheat-based breads and wholegrain breads grouped together, with spelt breads close to this group, whereas rye breads form a separated group (Fig. 4). As shown, there is variability among the rye breads across the Dim 1, determined for both rye percentage in their composition and fructan content. Moreover, the fructan content and the percentage of rye in bread products are correlated and opposite to total prolamin, total nitrogen, and Protein 2 contents (Fig. 4). The spelt breads are also grouped together in the PCA, and their variability depends on the spelt content, which is correlated with the total protein content (Fig. 4). Two breads made using 100% of durum wheat and 40% of tritordeum flour, respectively, are positioned close to Dim 2, to which the gluten content by R5 moAb contributes the most.

### Gluten and fructan allergens intake

**Fig. 5:**
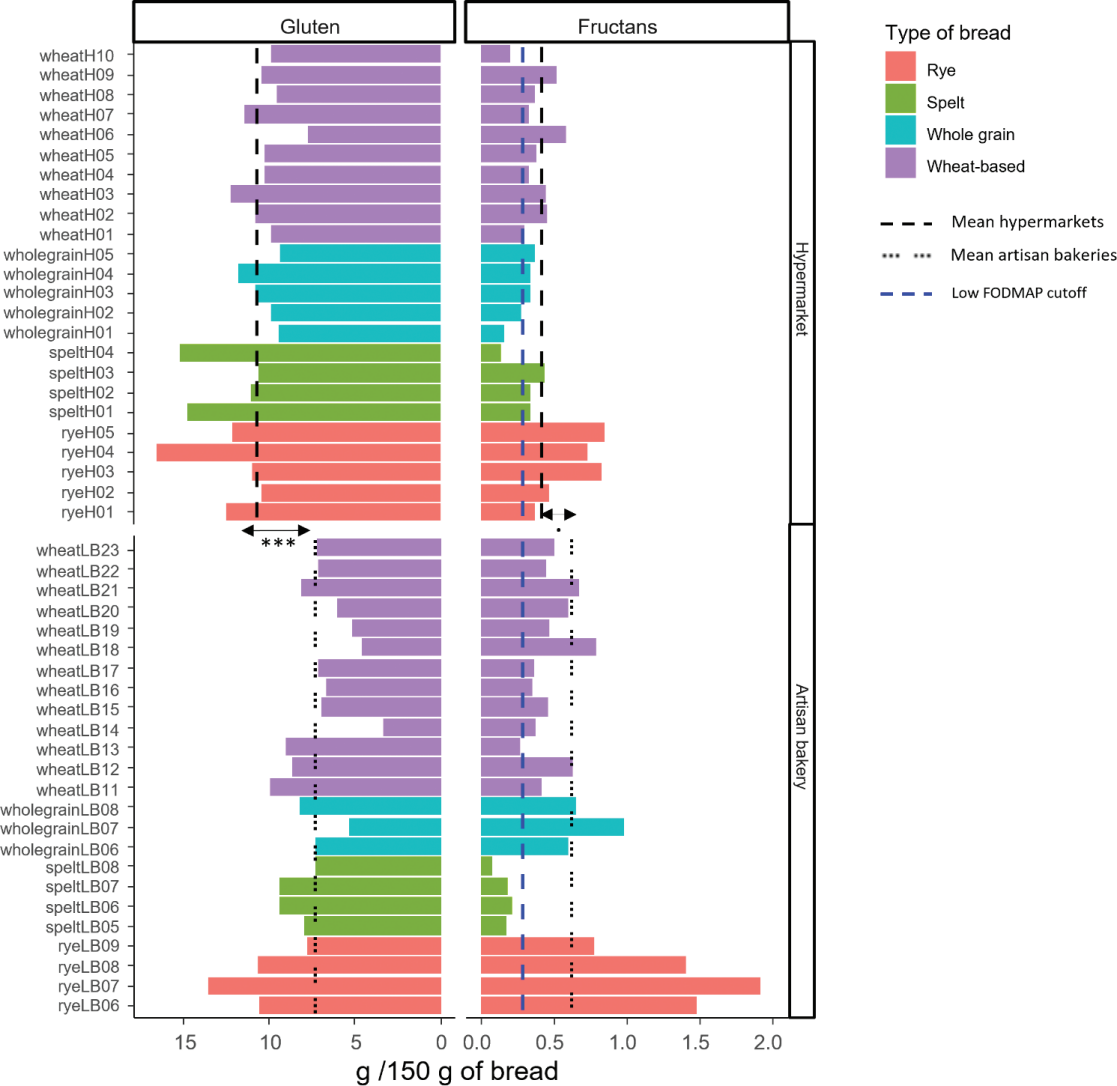
Total gluten and fructan intakes (g/150 g bread) for each bread. Mean value for each section is represented with black-dashed lines. Low-FODMAP cut-off value for bread wheat is denoted by the blue-dashed line (0.3) (27). Gluten intake was calculated as follows: gluten (ppm) × 150 bread (g) × 10E-6 × (1-water content [%]). Fructans intake was calculated as follows: fructan content (g/100 g FW bread) × 150 bread (g)/100 g. FW: fresh weight·, *P* ~ 0.05; ***, *P* < 0.001. Statistical significances were calculated by *t*-test and Mann–Whitney test for parametric and non-parametric data, respectively.

Based on the gluten (mg/kg) and fructan contents (g/100 g DW) of the different types of bread, and taking into account the water content of each type of bread, we have estimated the daily intake of these immunogenic compounds considering daily servings of 150 g of bread, which is the average daily bread consumption across several European countries and the United States (26). In the case of fructans, the cut-off to be considered as low FODMAP for wheat breads (0.3 g per serve) is also represented (27) (Fig. 5). As showed, the total gluten intake varies between type of bread. However, for both hypermarkets and artisan bakeries, rye breads showed the highest values of gluten consumption. Overall, hypermarkets’ breads have significantly higher mean (11.13) value of gluten intake per serving than artisan bakeries (7.78). The lower gluten intake in artisan breads is mainly showed in wheat-based and whole grain bread categories (Fig. 5). The bread made using 100% durum wheat flour (wheatLB14) is the one with the lowest gluten intake per serve (3.3 g), while the ryeH04 bread has the highest value for gluten intake (15.2 g) per serve, which is 4.6 times higher (Fig. 5). Interestingly, gluten intake of two spelt breads from hypermarkets had values (15.2 and 14.0, respectively) closer to that of rye breads with the highest gluten intake (Fig. 5).

In contrast to the gluten, for fructan intake, the mean value for hypermarkets’ breads (0.41) is lower, but not significant, than that of artisan bakeries’ (0.62). As for gluten, fructan intake was higher for rye-based breads, both for hypermarkets and artisan bakeries, 0.85 and 1.91 g, respectively (Fig. 5). For artisan breads, the mean value is highly influenced by rye and whole grain breads, which are made with 100% of these ingredients in most of the cases (Fig. 5). For spelt breads, fructan intake is higher in hypermarkets’ breads than that for artisan bakeries, while the range of variation in fructan intake was higher for wheat-based breads in artisan bakeries than in hypermarkets (Fig. 5). Considering the cut-off value for fructan intake, most of the breads present a fructan intake that beats the cut-off value, except spelt breads from artisan bakeries. Between wheat-based breads category, only wheatH10 and wheatLB13 showed values below this cut-off.

## Discussion

In recent years, there has been growing consumer concern about adverse reactions to wheat/gluten, and many consumers who want to reduce the wheat/gluten intake are adhering to a GFD. The direct consequence is an appreciable decrease in the consumption of bread and other wheat-based products. However, avoidance of gluten/wheat is strictly necessary in the case of CD, and for other pathologies, it should be up to the medical specialist to decide. Moreover, in the case of NCWS, the role of gluten is unclear, and other compounds such as fructans and ATIs were reported as the main causative agents (6, 28). In fact, many people with self-reported NCWS do not need to follow a totally GFD (29). In this work, we have evaluated the immunogenic potential of breads highly consumed in Spain and made using sourdough and long fermentation times (artisan bakeries) and those not using sourdough and short fermentation (hypermarkets). In addition, the daily intake of these immunogenic compounds has been assessed.

First, the extraction and quantification protocols by RP-HPLC were adapted to bread products, and times of elution compared to that of previous works (24). Two clear protein fractions, named as Protein 1 and Protein 2, and encompassing, respectively, ω-gliadins and HMW-glutenins, and α-, γ-gliadins, and LMW-glutenins were established. This allowed us to determine the total prolamin content in the bread and to compare it with the values obtained using the R5 monoclonal antibody. However, we did find a strong correlation of R5 neither with either Protein 1 or Protein 2 nor with the total prolamin content, with the best fitting obtained for spelt-based breads. We analyzed the gluten content using the ELISA R5 method because it is considered as a Type I analysis method by the Codex Alimentarius (CODEX STAN 118-1979) for the determination of gluten in food and is the method recommended by the Working Group on Prolamin Analysis and Toxicity (WGPAT). Moreover, it is widely used in the food industry for gluten content analysis. However, several studies mention that the R5 antibody underestimates or overestimates the gluten content, and that it produces a high variation in results in interlaboratory proficiency studies (30 31). Although the R5 moAb has been used for bread and wheat varieties screening (32, 33), this technique was not originally developed to perform measurements in sample with high-gluten content, which implies many dilutions during the analytical process that may lead to an error in gluten quantity estimation (34). These points might explain the low correlation between the RP-HPLC and R5 ELISA data for the breads analyzed in the present work. Moreover, the low correlation between both analytical methods may also be ligated to the relationship between the specificity/sensitivity of the R5 moAb and both the gluten source (rye, wheat, barley, and oat) and the gluten fraction (31), as shown for the rye breads samples, which will be discussed later.

The first main difference between breads provided by hypermarkets and artisan bakeries was the raw material used for bread elaboration. Artisan breads have 100% of the main ingredient for rye, spelt, and wholegrain breads in almost all cases, while breads sold in hypermarkets had highly variable content of the main ingredient, and bread wheat flour becomes also the most important ingredient for rye, spelt, and whole-grain breads. It is noteworthy that RP-HPLC chromatograms can help to detect mixtures of rye and wheat, as prolamins of both species show a very different pattern of elution. In addition to this, a second difference is the breadmaking process; all breads from artisan bakeries were made using sourdough and long fermentation times (minimum of 12 h) but not the hypermarket breads. This is an important issue as the use of sourdough and long fermentation times has been described as very effective in degrading FODMAPS (16), ATIs (35), and also gluten proteins (15), all compounds related in triggering adverse reactions to wheat/gluten.

Results reported in this work showed that except for rye breads, gluten content was significantly lower in breads from artisan bakeries. Rye breads had the highest gluten content in breads from both artisan bakeries and hypermarkets, while for the latter, spelt breads also had the highest gluten content. The fact that rye breads present a higher gluten content than wheat-based breads is probably because the R5 moAb was raised against rye secalins (36), being the overestimation of rye gluten – one of the R5 analytical method limitation (31). The lower gluten content in artisan bakeries compared to hypermarkets may be due to the longer fermentation times used in the production of artisan breads. The use of certain strains of lactobacilli and fungal proteases (37), as well as germinated-wheat sourdoughs (15), has been proposed as excellent alternatives to reduce the gluten content in bread, yielding reductions in gluten close to 20 mg/kg (37). In the case of the breads from artisan bakeries, long fermentation and sourdoughs were used, but addition of fungal proteases is not reported, and this may explain the moderate decreases in the gluten content compared to breads from hypermarkets with neither sourdough nor long fermentation times. This would indicate that sourdough and long fermentations would not be sufficient to degrade the gluten fractions present in flour. These results are in agreement with those reported by (34), where the use of sourdough and long fermentations reduces rye gluten, but fungal proteases were necessary to achieve extensive gluten degradation. Moreover, fermentations longer than 24 h appear to be less effective for protein degradation (34). The abundance of potentially allergenic proteins in flours from bread wheat, spelt and rye, and corresponding breads has also been reported (38). They compared the use of sourdough and yeast, but using short fermentation times instead. They reported that allergenic proteins were not selectively degraded during bread production, showing that the grain species have greater influence on bread proteome composition than fermentation processes (38).

The degradation of fructans in breads has been studied in sourdough fermentation, showing reductions of up to 75% in the fructan content of breads in comparison to that of flour (16). They also reported that yeast-only fermentation, without sourdough, also reduced significantly the fructan content, although this reduction was lower than that obtained with sourdough. However, our results show that except for spelt breads, artisan breads showed higher fructan contents than those from hypermarkets, despite being made with sourdough and long fermentations. In the case of rye, spelt, and whole grains, hypermarket breads were not produced using 100% flour from those cereals as the main ingredient. This would also explain why rye and whole grain breads from hypermarkets contain significantly less fructans than artisan bakeries. This is illustrated for one artisan bakery, whose rye bread is a mix of rye and wheat, and its content of fructan is more similar than that of the hypermarket breads. Fructan content in rye bread was previously reported ranging between 1.7 and 3.9% of total weight (39), being the highest source of fructans among all cereals (39). On the other hand, fructans are localized in the external part of the grain (40), and this could explain why the whole grain breads from artisan bakeries with 100% of this ingredient presented higher fructan content than hypermarkets’ ones. Whole grain has important health benefits, as recently reported that its consumption was linked to smaller increases in waist size, blood pressure, and blood sugar in middle- to older-aged adults, suggesting that whole grains may protect against heart disease (41).

Spelt-derived products are also gaining popularity as spelt wheat is considered an ancient grain, and these have been attributed with healthier properties (41, 42). In relation to the content of fructans and gluten, previous studies have pointed out clear differences for these compounds in spelt varieties (43). Fructan content in the grain is slightly lower in spelt wheat than that of bread and durum wheat, and rye (43, 44). In addition, gluten content and reactivity of gluten proteins, as determined by moAb R5 and G12, were higher than in bread wheat (45, 46). Overall, the results reported in this study confirm these differences for spelt breads, at least for artisan bakeries that used 100% of spelt flour. They contain fewer fructans than those containing rye, whole grain, and bread wheat. On the other hand, the gluten content of the spelt breads was higher than those made using whole grain and bread wheat.

In this study, the range of variation for gluten and fructan contents shown in breads from both artisan bakeries and hypermarkets would indicate that the raw material used is a principal factor to develop low-content, low-stimulatory breads. For example, some breads from artisan bakeries were elaborated using a high percentage of durum wheat or tritordeum flour, which are the breads with the lowest immunogenic potential. Both tritordeum and durum wheat lack the D genome, which contains the α-gliadins harboring the 33-mer peptide (47), one of the most immunogenic peptide described so far in relation to CD (48). In fact, tritordeum breads were well tolerated by NCWS patients with important benefits in gut microbiota (49). In the case of fructans, the range of variation was higher for artisan bakeries than that from hypermarkets, with clear and significant differences between the different types of breads. In contrast, the breads from hypermarkets were much more homogeneous in the fructan content, and only those containing rye are significantly different from the rest, except for wheat and whole-grain breads comparison, indicating that the bread wheat flour (used to complete different breads) has a very important normalizing effect.

In order to find out differences in the daily intake of gluten and fructans, we considered a serving size of 150 g of bread per day. Our results indicate that there is a huge variation for gluten and fructans that are consumed daily depending on the type of bread and its origin. The gluten consumed ranged from 3.3 to 16.5 g per serving size. The lower values correspond to breads made using 100% and 50% durum wheat flour, and 40% tritordeum flour. In contrast, the higher values for gluten consumed correspond to spelt and rye breads sold in hypermarkets. The values for low and high gluten intake reported in this work are close to those used in (50), where they compared the effects of a low-gluten diet with a high-gluten diet in healthy people. They reported that a low-gluten diet induces moderate changes in the intestinal microbiome, reduces fasting and postprandial hydrogen exhalation, and leads to improvements in self-reported bloating. Moreover, a decrease in body weight following the low-gluten was also observed in comparison to the high-gluten diet (50). However, those changes were also linked to shifts in the fermentation of complex carbohydrates, and therefore, it could not be addressed solely to the low-gluten. However, significant beneficial changes in the microbiota were also observed in NCWS patients who followed a diet with a bread made using a very low-gluten RNAi wheat flour (51).

Collectively, the mean of gluten intake per serving size (g/150 g of bread) in hypermarkets’ breads (11.2) was higher than artisan bakeries’ ones (7.8), being an interesting key point in consumer decision at the time to choose. However, the mean for fructan intake per serving size (g/150 g of bread). was non-significantly higher for artisan breads (0.62) than for hypermarkets one (0.41). Interestingly, the fructan intake from artisan bakeries corresponding to all spelt breads remaining below the threshold established for low-FODMAP diet (27), while spelt breads from hypermarkets had higher fructan content than this cutoff except for one of them, which had the highest percentage of spelt between hypermarket breads. However, the cutoff was previously established using the K-FRUC HK kit to measure the fructan content (27). The K-FRUC kit used in the present work was reported more suitable for fructan determination in cereal samples with low fructan content and high non-targeted carbohydrates (52, 53), whereas the K-FRUC HK kit might overestimates fructans in bread (53).

## Conclusions

The results reported in the present work reveal that there is a wide range of variation for the content of the two major immunogenic/allergenic compounds (gluten and fructan) in breads from artisan bakeries and hypermarkets. In general, breads from artisan bakeries showed a lower amount of gluten but a higher amount of fructans than that of hypermarkets. The use of sourdough and long fermentation times in artisan bakeries could be responsible for the lower gluten content. The raw material used is a key factor to explain the differences for the immunogenic/allergenic compounds, both within each category and for the comparison between artisan bakeries and hypermarkets. Breads elaborated using durum wheat and tritordeum flour provided breads with the lowest immunogenic potential, while rye flour had highest. All these differences are reflected in the amount of gluten and fructans that consumers intake daily, with differences of up to 4.5 and 20 times for gluten and fructan, respectively. We obtained a low correlation between the RP-HPLC prolamin content and the gluten content measured by the R5 ELISA test, mainly for rye breads.
